# Changes in the Prevalence, Treatment and Control of Hypertension in Germany? A Clinical-Epidemiological Study of 50.000 Primary Care Patients

**DOI:** 10.1371/journal.pone.0052229

**Published:** 2012-12-28

**Authors:** Alexander Michael Labeit, Jens Klotsche, Lars Pieper, David Pittrow, Franziska Einsle, Günter Karl Stalla, Hendrik Lehnert, Sigmund Silber, Andreas Michael Zeiher, Winfried März, Martin Wehling, Hans-Ulrich Wittchen

**Affiliations:** 1 Clinical Pharmacology Mannheim, Institute of Experimental and Clinical Pharmacology and Toxicology, Medical Faculty Mannheim of the University of Heidelberg, Heidelberg, Germany; 2 Department of Health Sciences, University of Leicester, Leicester, United Kingdom; 3 Institute of Clinical Psychology and Psychotherapy and Center of Clinical Epidemiology and Longitudinal Studies, TU Dresden, Dresden, Germany; 4 Max Planck Institute of Psychiatry, Munich, Germany; 5 Department of Internal Medicine I, University of Lübeck, Lübeck, Germany; 6 Private Cardiology Practice and Clinic, Munich, Germany; 7 Department of Internal Medicine III, Cardiology, Goethe-University Frankfurt, Frankfurt, Germany; 8 Synlab Academy, Synlab Services GmbH, Mannheim, Germany; 9 Mannheim Institute of Public Health, Rupertus Carola University of Heidelberg, Medical Faculty Mannheim, Mannheim, Germany; 10 Clinical Institute of Medical and Chemical Laboratory Diagnostics, Medical University of Graz, Graz, Austria; University of Oxford, United Kingdom

## Abstract

**Introduction:**

Medical societies have developed guidelines for the detection, treatment and control of hypertension (HTN). Our analysis assessed the extent to which such guidelines were implemented in Germany in 2003 and 2001.

**Methods:**

Using standardized clinical diagnostic and treatment appraisal forms, blood pressure levels and patient questionnaires for 55,518 participants from the cross-sectional Targets and Essential Data for Commitment of Treatment (DETECT) study (2003) were analyzed. Physician’s diagnosis of hypertension (HTN_doc_) was defined as coding hypertension in the clinical appraisal questionnaire. Alternative definitions used were physician’s diagnosis or the patient’s self-reported diagnosis of hypertension (HTN_doc,pat_), physician’s or patient’s self-reported diagnosis or a BP measurement with a systolic BP≥140 mmHg and/or a diastolic BP≥90 (HTN_doc,pat,bp_) and diagnosis according to the National Health and Nutrition Examination Survey (HTN_NHANES_). The results were compared with the similar German HYDRA study to examine whether changes had occurred in diagnosis, treatment and adequate blood pressure control (BP below 140/90 mmHg) since 2001. Factors associated with pharmacotherapy and control were determined.

**Results:**

The overall prevalence rate for hypertension was 35.5% according to HTN_doc_ and 56.0% according to NHANES criteria. Among those defined by NHANES criteria, treatment and control rates were 56.0% and 20.3% in 2003, and these rates had improved from 55.3% and 18.0% in 2001. Significant predictors of receiving antihypertensive medication were: increasing age, female sex, obesity, previous myocardial infarction and the prevalence of comorbid conditions such as coronary heart disease (CHD), hyperlipidemia and diabetes mellitus (DM). Significant positive predictors of adequate blood pressure control were CHD and antihypertensive medication. Inadequate control was associated with increasing age, male sex and obesity.

**Conclusions:**

Rates of treated and controlled hypertension according to NHANES criteria in DETECT remained low between 2001 and 2003, although there was some minor improvement.

## Introduction

Hypertension (HTN) is a common cause for patient contacts with primary physicians, and there is evidence of suboptimal treatment and inadequate blood pressure control [1]. In Germany the control status of hypertensive patients in the community [2] and in primary care [3] has been repeatedly and consistently found to be lower in comparison with other European countries and the USA [4], [5]. Prompted by these data and because of the medical consequences and economic impact of hypertension, considerable national and international activities targeting improved recognition, treatment and control have been initiated [6], [7]. Against this background, re-examination of the recorded prevalence and control of hypertension is essential to monitor changes that might have occurred as a result of enhanced prevention and treatment activities. Furthermore, a focus on primary care is appropriate, because of the essential role of primary care physicians as gatekeepers for hypertension management in the German health care system [8].

Patients with hypertension alone have an increased risk for cardiovascular morbidity and mortality [9] and hypertensive patients with DM and/or metabolic syndrome as comorbidities have even an increased risk for cardiovascular morbidity and mortality compared to patients with hypertension alone [10], [11]. It has been shown by meta-analyses and different large-scale studies that strict control of risk factors including cardiovascular disease, DM, hyperlipidemia and smoking status in patients with hypertension can effectively reduce cardiovascular mortality among patients with hypertension [12]. A meta-analysis showed that type-2 diabetic patients might benefit more from lower blood pressure goals than non-diabetic patients with hypertension [13]. Consequently, national and international medical societies have developed clear recommendations and guidelines for hypertension and risk factor management and respective therapeutic targets [14]. For example, the Seventh Report of the Joint National Commission on Prevention, Detection, Evaluation and Treatment of High blood pressure guidelines (JNC-7) recommend blood pressure targets with systolic blood pressure levels below 140 mmHg and diastolic blood pressure levels below 90 mmHg, while for patients with comorbidities the blood pressure (BP) recommendation has been set at 130/80 mmHg [12]. In hypertensive patients with DM, tight control of blood pressure is of particular importance and intense non-pharmacological and pharmacological interventions should be implemented to lower blood pressure to below 130/80 mmHg [15]. In contrast to the clear recommendations and guidelines of medical scientific societies, there is increasing evidence for a disparity between scientifically grounded treatment targets and the actual implementation of guidelines by physicians in Germany [16].

Our analysis compares the results of the DETECT study with 55,518 participants in 2003 for the estimated prevalence rates using different definitions for diagnosis of hypertension and compares treatment and control rates with the results of the Hypertension and Diabetes Risk Screening and Awareness Study (HYDRA) study [3] with 45,125 participants in 2001 and also comparisons with other German and international studies are made. At the same time the determinants of pharmacotherapy and adequate blood pressure control including among high-risk patients with comorbidities are examined. We used the criteria of the JNC 7 guidelines for the classification of the severity of hypertension: normal BP for a systolic BP<120 mmHg and a diastolic BP<80 mmHg; prehypertension for a systolic BP between 120 to 139 mmHg and/or diastolic BP between 80 to 89 mmHg; stage 1 hypertension for a systolic BP between 140 and 159 and/or a diastolic BP between 99 and 90 mmHg; stage 2 hypertension for a systolic BP≥160 mmHg and/or a diastolic BP≥100 mmHg. These categories are almost identical to those suggested by the European Society of Hypertension/European Society for Cardiology (ESH/ESC) [12]. If the systolic and diastolic BP would have led to a different categorisation for hypertension the higher category was chosen.

## Methods

DETECT (**D**iabetes Cardiovascular Risk-**E**valuation: **T**argets and **E**ssential **D**ata for **C**ommitment of **T**reatment) was initiated to estimate the prevalence and comorbidity patterns of primary care patients and to evaluate new strategies for the prevention and early detection of chronic diseases [17], [18]. The DETECT study was designed to evaluate the impact of characteristics of the patient and the physician on diagnosis, treatment and control of hypertension. It is a large-scale, cross-sectional study with longitudinal follow-up of a subcohort and nationally representative of primary care situation in Germany [3]. Sampling and data collection for the DETECT study were very similar to the HYDRA study and based on a nationwide sample of physicians with primary care functions (medical practitioners, generalists, general internists). More information about the DETECT study can be found on http://www.detect-studie.de and about the HYDRA study on http://www.hydra-studie.de. HYDRA was a precursor study of DETECT with identical methods of sampling, design and also assessment of hypertension [19]. All patients consulting a practice for whatever reason on 16^th^ or 18^th^ September 2003 were included in the DETECT study. The sampling was based on 1060 regional segments in Germany (according to the criteria of the Institute for Medical Statistics, Frankfurt am Main, Germany), 7053 primary care physicians being randomly selected as a sample from all 64707 physicians who had been working as primary care physicians in Germany in 2003 [3]. Following a pre-study to determine characteristics of physicians and settings, the cross-sectional baseline study in the year 2003 was based on a sample of 55519 unselected adult patients, who were examined in a total of 3188 nationally representative primary care offices (73% general practitioners and 27% internists). Participating patients completed a standardized questionnaire regarding sociodemographic and lifestyle factors. Physicians completed a standardized questionnaire asking for information about known or newly diagnosed medical conditions and an evaluation of the clinical and therapeutic profiles of the patients. Information about prescribed anti-hypertensive medication and results of the current examination (e.g. blood lipid levels, blood pressure measurements according to the JNC 7 report [20]) were also provided by the physician. For the analysis baseline data of the DETECT study for the year 2003 were used. Age groups were defined as follows: 18–29, 30–44, 45–59, 60–74 and 75+ years.

### Ethics Statement

The DETECT study was approved by the ethics committee of the Dresden University of Technology (TU Dresden). Written informed consent was received from all participants.

### Diagnostic Conventions

In order to allow a comparison of the DETECT study specifically with the previous HYDRA study and further related studies in Germany, this paper applies the methods used and described in a previous study for the measurement of prevalence of hypertension [3]. In DETECT, blood pressure measurements were taken in the physicians’ offices as routine part of the physical examination and were done by indirect cuff sphygmomanometry after a 10-minute of rest in a sitting position according to the international guidelines [20]. In both studies (HYDRA and DETECT) blood pressure was only measured once. Prevalence rates of hypertension were examined using the following definitions which has also used in the HYDRA study as precursor study: a) physician’s diagnosis of hypertension as coded in the clinical appraisal questionnaire (HTN_doc_); b) physician’s diagnosis *or* the patient’s self-reported diagnosis of hypertension (HTN_doc,pat_); c) physician’s *or* patient’s self-reported diagnosis *or* a BP measurement with a systolic BP≥140 mmHg *and/or* a diastolic BP≥90 (HTN_doc,pat,bp_); d) diagnosis according to the National Health and Nutrition Examination Survey (HTN_NHANES_) [21], i.e. a measured BP≥140/90 mmHg *or* treatment with antihypertensive drugs, irrespective of the doctor’s or patient’s self-reported diagnosis. For the estimation of the proportion of diagnosed patients (<140/90 mmHg) we used the HTN_NHANES_ criterion for defining hypertension [21], [22]. These patients were defined as treated if they received antihypertensive drugs and the following drug classes were included: ACE inhibitors, AT1-antagonists, beta-blockers, calcium channel blockers and diuretics. These patients were defined as controlled if blood pressure was below 140/90 mmHg.

### Cardiovascular Comorbidity

The physicians’ questionnaire explicitly asked for the existence of 28 specified medical diagnoses and also allowed for adding further diagnoses. CHD, DM and hyperlipidemia were exclusively defined as physicians’ based diagnoses (newly diagnosed or previously known) [18]. The physicians’ questionnaire contained information about previous myocardial infarction and family history of myocardial infarction.

### Cardiovascular Risk Factors

The patients’ questionnaire contained information about physical activity and smoking status as cardiovascular risk factors. Physical activity was classified as either more than or equal to 2 hours per week versus physical activity with less than 2 hours per week on average (e.g. gardening, cycling, walking or other sports). Smoking status of the participants was defined as use of any tobacco product in the past 4 weeks and categorized as yes/no. BMI was categorized into three groups: <25 kg/m^2^ (normal), 25 to <30 kg/m^2^ (overweight) and ≥30 kg/m^2^ (obesity). Waist circumference (WC) was categorized into the following groups: ≤94 cm for males and ≤80 cm for females as not overweight, >94 cm and ≤102 cm for males and >80 cm and ≤88 cm for females as pre-obese, >102 cm for males and 88 cm for females as obese.

### Statistical Analyses

DETECT study participants were analyzed with respect to socio-demographic, comorbidities and cardiovascular lifestyle risk factors. Gender and age-group specific estimates of prevalence rates of hypertension were determined applying alternative definitions of hypertension in unselected primary care patients. Also the prevalence of hypertension according to JNC 7 categories was analyzed. Determinants associated with pharmacotherapy and adequate BP control of hypertension such as socio-demographic comorbidities and cardiovascular lifestyle risk factors were evaluated using multiple logistic regression. Education in years was included in the models as potentially explanatory factor. The patients were clustered by primary care units and to account for the clustered structure of the data, robust standard errors were estimated by the Huber-White Matrix. Data were weighted for non-response and attrition during the recruitment phase. Categorical data were presented as absolute frequencies (n) and weighted percentages (%w), metric variables by weighted mean and standard deviation. A two-sided alpha-level of 0.05 was chosen as criterion for statistical significance. All statistical analyses were conducted using STATA 11.2 (Stat Corp. 2009, College Station, Texas, USA).

## Results

Table 1 presents descriptive statistics for sociodemographic and baseline variables for the 55,518 participants of the DETECT study in 2003. The average systolic BP was 131.7 mmHg and diastolic BP was 79.9 mmHg. Of the participants of the DETECT study, 12.1% had physician diagnosed DM, 29.5% hyperlipidemia, 14.7% CHD and 4.2% previous myocardial infarction. Physician based diagnosis of hypertension (HTN_doc_) was present in 35.5% of all participants (see table 2). The prevalence rate of physician diagnosed hypertension increased from the youngest age group (18 to 29 years) to the oldest one (75 years and above) from 2.9% from to 62.6%. Physician’s diagnosis or the patient’s self-reported diagnosis of hypertension (HTN_doc,pat_) showed slightly higher prevalence rates: 41.8% in all participants, 6.4% in the youngest age group and 69.5% in the oldest age group. Physician’s or patient’s self-reported diagnosis or a BP measurement with a systolic BP≥140 mmHg and/or a diastolic BP≥90 (HTN_doc,pat,bp_) prevalence rates increased to 55.2% in all participants and were 15.4% and 82.1% in the youngest and oldest age group, respectively. Prevalence rates for hypertension diagnosis according to the National Health and Nutrition Examination Survey (HTN_NHANES_) were in a similar range as for HTN_doc,pat,bp_: 56.0% in all participants, 13.3% in the youngest and 86.8% in the oldest age group. Comparing the estimated prevalence rates for HTN_doc_, HTN_doc,pat_, HTN_doc,pat,bp_ and HTN_NHANES,_ the largest differences measured as ratio of 2 estimated prevalence rates were seen in the youngest age group comparing HTN_doc_ and HTN_NHANES_. The biggest percent difference in absolute number was present in the oldest age group comparing HTN_doc_ to HTN_NHANES_. Limitations of the DETECT study exist, because there was only single measurement of blood pressure in a primary care setting done. Some patients have been excluded from the study, because of time restrictions of the physician or no clinical assessment was possible [23]. Several measurements are recommended and prevalence of hypertension is dependent from the setting and inclusion criteria of patients [18].

**Table 1:** Sample characteristics of DETECT study participants.

	Total		Male		Female	
	(N = 55,518)		(N = 22,679)		(N = 32,839)	
	N	%w	N	%w	N	%w
Age (mean/sd)	53.8/17.4		54.5/16.8		53.3/17.8	
18–29 years	6,031	11	2,249	9.9	3,782	11.7
30–44 years	11,525	21	4,328	19.5	7,197	22.1
45–59 years	13,555	24.3	5,599	24.5	7,956	24.1
60–74 years	17,934	31.9	8,087	35.3	9,847	29.5
75+ years	6,473	11.9	2,416	10.9	4,057	12.6
Systolic blood pressure, (mean/sd)	131.7/18.4		133.8/17.3		130.3/19.0	
Diastolic blood pressure, (mean/sd)	79.9/9.9		80.8/9.8		79.2/10.0	
Years of schooling, (mean/sd)	10.1/2.1		10.2/2.2		10.0/2.0	
BMI (mean/sd)	26.8/5.3		27.3/4.7		26.5/5.6	
<25 kg/m^2^	21,520	40.2	7,037	32.1	14,483	45.8
25–29.9 kg/m^2^	20,383	37.3	10,201	45.8	10,182	31.5
30+ kg/m^2^	12,491	22.5	4,986	22.2	7,505	22.7
WC (mean/sd)	93.8/15.6		99.8/13.8		89.7/15.4	
≤94 cm (male) ≤80 cm (female)	15,595	31.9	6,667	33.6	8,928	30.7
>94 cm–≤102 cm (male) >80 cm–≤88 cm (female)	10,729	21.9	5,245	26.4	5,484	18.9
>102 cm (male) >88 cm (female)	22,593	46.2	7,930	40	14,663	50.4
Current smoker	13,504	26.1	6,186	29.2	7,318	24
Physical activity <2 hours/week	16,265	34.3	5,902	30.3	10,363	37.2
*Comorbidities*						
CHD	8,465	14.7	4,127	17.6	4,338	12.8
Hyperlipidemia	16,178	29.5	7,311	32.4	8,867	27.5
Diabetes mellitus	6,895	12.1	3,969	17.2	2,926	8.6
Myocardial infarction	2,326	4.2	1,649	7.3	677	2.1

N = 55,518, %w = weighted percentages; BMI = body mass index in kg/m2.

Number of valid observations: Blood pressure n = 53,337 (96.1%); Years of schooling n = 53,406 (96.2%); Body mass index.

n = 54,393 (98.0%); Waist circumference n = 48,918 (88.1%); Smoking status n = 52,589 (94.7%); Physical activity n = 48,249 (86.9%).

**Table 2:** Prevalence rates of hypertension by age, sex and type of diagnosis.

		no HTN_doc_	HTN_doc_		no HTN_doc/Pat_	HTN_doc/Pat_		no HTN_doc/Pat/bp_	HTN_doc/Pat/bp_		no HTN_NNANES_^†^	HTN_NHANES_^†^	
		N	N	%w	N	N	%w	N	N	%w	N	N	%w
Total	Total	35,354	20,164	35.5	31,858	23,660	41.8	24,569	30,949	55.2	23,148	30,189	56.0
	Female	21,539	11,300	33.6	19,667	13,172	39.3	15,701	17,138	51.6	14,969	16,620	52.0
	Male	13,815	8,864	38.3	12,191	10,488	45.5	8,868	13,811	60.4	8,179	13,569	61.9
18–29 years	Total	5,854	177	2.9	5,634	397	6.4	5,099	932	15.4	5,017	771	13.3
	Female	3,693	89	2.3	3,586	196	5.0	3,324	458	12.2	3,258	372	10.3
	Male	2,161	88	4.0	2,048	201	8.8	1,775	474	20.8	1,759	399	18.3
30–44 years	Total	10,291	1,234	10.1	9,726	1,799	14.9	8,192	3,333	28.3	8,014	3,056	27.1
	Female	6,551	646	8.5	6,250	947	12.6	5,499	1,698	23.1	5,369	1,558	22.1
	Male	3,740	588	12.7	3,476	852	18.8	2,693	1,635	37.0	2,645	1,498	35.5
45–59 years	Total	9,068	4,487	32.0	8,113	5,442	39.1	5,992	7,563	55.1	5,744	7,291	55.1
	Female	5,559	2,397	29.0	5,062	2,894	35.3	3,891	4,065	50.3	3,754	3,908	50.2
	Male	3,509	2,090	36.3	3,051	2,548	44.5	2,101	3,498	61.8	1,990	3,383	62.2
60–74 years	Total	7,778	10,156	56.0	6,460	11,474	63.5	4,149	13,785	76.7	3,569	13,664	79.0
	Female	4,294	5,553	55.7	3,590	6,257	63.0	2,321	7,526	76.2	2,104	7,375	77.5
	Male	3,484	4,603	56.2	2,870	5,217	64.0	1,828	6,259	77.3	1,465	6,289	80.9
≥75 years	Total	2,363	4,110	62.6	1,925	4,548	69.5	1,137	5,336	82.1	804	5,407	86.8
	Female	1,442	2,615	63.3	1,179	2,878	70.0	666	3,391	83.1	484	3,407	87.1
	Male	921	1,495	61.2	746	1,670	68.8	471	1,945	80.5	320	2,000	86.1

N = 55,518, † N = 53,337 patients with valid blood pressure assessment; %w = weighted percentages HTNdoc: doctor’s diagnosis; HTNdoc/pat: doctor’s or patient’s diagnosis; HTNdoc/pat/bp: doctor’s or patient’s diagnosis or blood pressure 140/90 mmHg; HTNHANES: blood pressure >140/90 mmHg or receiving antihypertensive therapy.

The categories of the measured BP values according to the JNC 7 classification are shown in table 3 stratified by age and sex. 14.9% of the participants had normal blood pressure, 44.6% prehypertension, 28.8% stage 1 hypertension and 11.6% stage 2 hypertension. The prevalence rates of hypertension stages 1 and 2 increased together with age. In participants between age 18 and 29 prevalence rates were 10.2% and 1.8%, in patients above 75 years of age prevalence rates were 41.2% and 18.7%, respectively. The mean systolic BP increased continuously with age up to 140.6 mmHg in participants above 75 years of age and diastolic BP increased up to 81.6 mmHg in the age group 60–74 years, but decreased to 79.9 mmHg again in the oldest age group.

**Table 3:** Prevalence of JNC 7 Blood Pressure Categories.

		BP<120/80 mmHg		BP 120–139/80–89 mmHg		BP 140–159/90–99 mmHg		BP≥160/100 mmHg	
		N	%w	N	%w	N	%w	N	%w
Total†	Total	7,839	14.9	23,890	44.6	15,489	28.8	6,119	11.6
	Female	5,729	18.5	13,894	43.8	8,486	26.6	3,480	11.2
	Male	2,110	9.8	9,996	45.8	7,003	32.1	2,639	12.3
18–29 years	Total	2,114	36.8	2,982	51.3	584	10.2	108	1.8
	Female	1,608	44.7	1,699	46.2	284	8.0	39	1.0
	Male	506	23.1	1,283	59.9	300	13.8	69	3.2
30–44 years	Total	2,681	24.6	5,882	52.9	1,935	17.3	572	5.2
	Female	2,155	31.5	3,539	50.9	975	13.9	258	3.7
	Male	526	13.1	2,343	56.3	960	22.9	314	7.7
45–59 years	Total	1,630	12.8	6,089	46.6	3,880	29.5	1,436	11.1
	Female	1,216	16.2	3,623	47.2	2,091	27.0	732	9.6
	Male	414	7.9	2,466	45.9	1,789	33.0	704	13.2
60–74 years	Total	1,081	6.3	6,768	39.1	6,519	37.7	2,865	17.0
	Female	583	6.2	3,723	39.2	3,518	36.8	1,655	17.8
	Male	498	6.5	3,045	38.9	3,001	38.8	1,210	15.9
≥75 years	Total	333	5.4	2,169	34.8	2,571	41.2	1,138	18.7
	Female	167	4.3	1,310	33.6	1,618	41.1	796	21.0
	Male	166	7.2	859	36.7	953	41.3	342	14.8

† N = 53,337 patients with valid blood pressure assessment; %w = weighted percentages; BP = blood pressure.

### Diagnosis, Treatment and Control Rates in the DETECT Study

Across all age groups 61.5% of HTN_NHANES_ cases were diagnosed (coded in the clinical appraisal) by the physicians. In the youngest there was the smallest proportion of diagnosed HTN_NHANES_ cases (17.4% only). In the age group 75+ the fraction of physician diagnosed cases was highest (71.0%). The rates of HTN_NHANES_ cases, which were diagnosed and also treated by physicians increased with higher age: 12.1% in the youngest age group and 65.7% in the oldest age group. Among HTN_NHANES_ cases the proportion of patients with adequate blood pressure control was 21.4% across all groups; it was 5.8% in the youngest and 23.3% in the oldest age group.

### Comparison between HYDRA and DETECT Study

**Figure 1:**
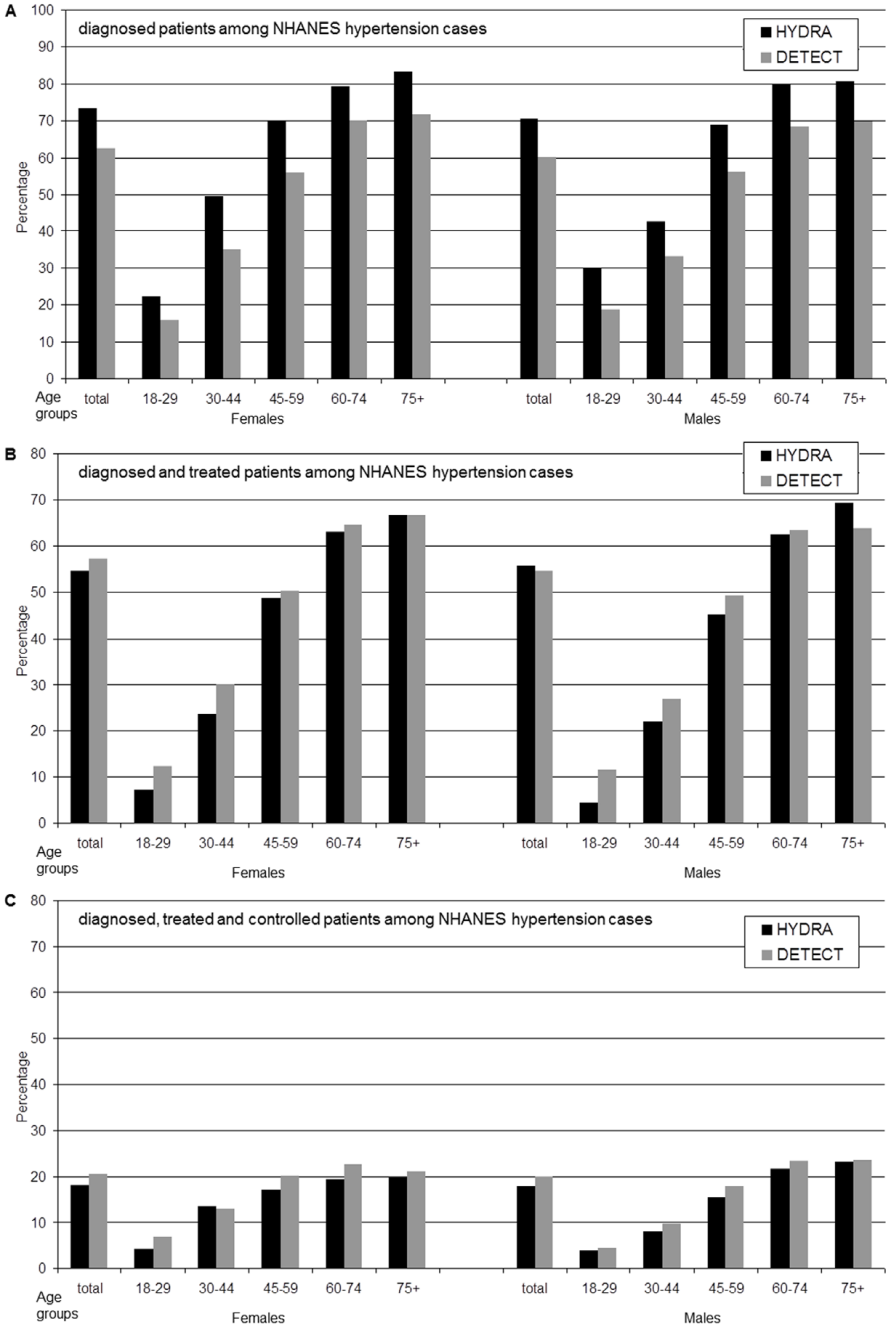
Diagnosis, treatment and control rates among NHANES hypertension cases in DETECT and HYDRA study. Comparison between DETECT study (N = 30189) and HYDRA study (N = 22744) for diagnosis, treatment and control rates. Diagnosed = diagnosed by physician among NHANES cases; diagnosed+treated = diagnosed by physician and treated by antihypertensive drugs among NHANES cases; diagnosed+treated+controlled = diagnosed by physician, treated by antihypertensive drugs and blood pressure <140/90 mmHg among NHANES cases. A comparison between both studies reveals that the proportion of diagnosed patients among NHANES hypertension cases was lower in the first study for all age groups above age 30 for men and women (p<0.05). Small (significant) improvements in rates of diagnosed and treated hypertension were found in females 30–44 years of age and in males 30–44 and 45–59 years of age (p<0.05). Small (significant) improvements in rates of diagnosed, treated and controlled hypertension were found in females 60–64 years of age and in males 30–44 and 45–59 years of age (p<0.05).

The comparison presented in figure 1 reveals that the proportion of diagnosed patients was lower in the DETECT study in comparison to the HYDRA study. These changes are consistent, and are significant (p<0.05) in all age groups above age 30 for men and women. The comparison between these studies shows also indications of, albeit small, improvements in rates of treated and controlled hypertension. The changes are consistent, although not significant in all gender and age group comparisons. Significant improvements (p<0.05) in the rates of treatment were found in females 30–44 years of age and in males 30–44 and 45–59 years of age. Rates of antihypertensive drug therapy decreased significantly in male patients aged 75 years and older. Adequate BP control was significantly more common in older female patients (age group 60+ years).

### Factors Associated with Pharmacotherapy

Table 4 shows factors associated with pharmacotherapy for hypertension. Patients in the oldest age group were approximately tenfold more likely to take antihypertensive drugs (OR 10.58, 95%-CI 8.36–13.39). Further factors associated with receiving antihypertensive medication in NHANES based cases were female sex (OR 1.18, 95%-CI 1.10–1.27), BMI above 30 kg/m^2^ (OR 1.72, 95%-CI 1.57–1.89), a history of CHD (OR 4.64, 95%-CI 3.92–5.50), a physician based diagnosis of hyperlipidemia (OR 1.66, 95%-CI 1.54–1.79), DM (OR 1.98, 95%-CI 1.79–2.19) or previous myocardial infarction (OR 2.27, 95%-CI 1.60–3.21). An alternative specification with waist circumference (reference category: not overweight) instead of BMI as a measure of cardiovascular risk gave very similar results. Smoking was negatively associated with receiving antihypertensive medication (OR 0.79, 95%-CI 0.72–0.86).

**Table 4:** Determinants of pharmacotherapy among NHANES hypertension cases.

		Antihypertensive treatment (N = 21,323)					
	All	With treatment		Unadjusted		Adjusted†	
	N	N	%w	OR#	95% CI	OR†	95% CI
Male gender	12,941	9,464	72.3	(reference)		(reference)	
Female gender	15,876	11,859	73.9	1.08*	1.02–1.14	1.18*	1.10–1.27
*Age in years*							
18–29	682	146	20	(reference)		(reference)	
30–44	2,811	1,194	40.8	2.75*	2.24–3.39	2.08*	1.67–2.59
45–59	6,903	4,604	65.6	7.62*	6.24–9.30	4.57*	3.70–5.65
60–74	13,202	10,827	81.4	17.57*	14.42–21.40	8.19*	6.61–10.16
≥75	5,219	4,552	86.5	25.68*	20.81–31.68	10.58*	8.36–13.39
Years of schooling, (mean/sd)‡		9.4/2.0		0.87*	0.86–0.88	0.98	0.97–1.00
*BMI*							
<25 kg/m^2^	7,095	4,745	66.3	(reference)		(reference)	
25–29.9 kg/m^2^	12,169	9,008	73.4	1.41*	1.32–1.51	1.24*	1.14–1.35
30+ kg/m^2^	9,186	7,283	78.2	1.82*	1.69–1.96	1.72*	1.57–1.89
Current non-smoker	22,809	17,536	76.1	(reference)		(reference)	
Current smoker	4,912	2,952	59.6	0.46*	0.43–0.50	0.79*	0.72–0.86
Physical activity ≥2 hours/week	16,422	12,025	72.2	(reference)		(reference)	
Physical activity <2 hours/week	7,903	5,660	70.9	0.94*	0.88–1.00	1.02	0.94–1.09
*Comorbidities*							
no CHD	22,829	15,639	67.6	(reference)		(reference)	
CHD	5,988	5,684	94.9	8.83*	7.80–9.99	4.64*	3.92–5.50
no Hyperlipidemia	16,825	11,274	65.8	(reference)		(reference)	
Hyperlipidemia	11,992	10,049	83.2	2.56*	2.41–2.72	1.66*	1.54–1.79
no Diabetes mellitus	21,814	15,144	68.6	(reference)		(reference)	
Diabetes mellitus	7,003	6,179	87.8	3.28*	3.02–3.56	1.98*	1.79–2.19
no Myocardial infarction	26,719	19,287	71.3	(reference)		(reference)	
Myocardial infarction	2,098	2,036	96.9	12.66*	9.73–16.47	2.27*	1.60–3.21

N = 30,189; %w = weighted percentages; OR = Odds Ratio estimated by logistic regression; 95% CI = 95% confidence interval.

# unadjusted OR;

† adjusted OR from multivariate analyses;

* significant on 5% level.

‡ OR for increase of 1 year.

### Factors Associated with Blood Pressure Control

In HTN_doc_ cases, the factors associated with adequate blood pressure control (less than 140/90 mmHg) were female sex (OR 1.16, 95%-CI 1.07–1.25), the presence of CHD (OR 1.25, 95%-CI 1.13–1.39), previous myocardial infarction (OR 1.76, 95%-CI 1.50–2.05) and antihypertensive medication (OR 1.41, 95%-CI 1.23–1.61) (table 5). With increasing age the likelihood of adequate blood pressure control was reduced (OR 0.57, 95%-CI 0.39–0.82 for the highest age group) and also for a BMI above 30 kg/m^2^ (OR 0.67, 95%-CI 0.61–0.74). An alternative specification with waist circumference (reference category: not overweight) instead of BMI as a measure of cardiovascular risk gave also very similar results in this regression. Comorbidities (hyperlipidemia, DM and lifestyle factors) such as physical activity and smoking status) had no effect on blood pressure control.

**Table 5:** Determinants of adequate blood pressure control among physician diagnosed hypertension cases.

			Adequate blood pressure control (N = 6989)					
		All	Adequate control		Unadjusted		Adjusted†	
		N	N	%w	OR#	95% CI	OR†	95% CI
Male gender		8,570	3,085	35.5	(reference)		(reference)	
Female gender		10,959	3,904	35.3	0.99	0.93–1.05	1.16*	1.07–1.25
*Age in years*								
18–29		168	77	45.3	(reference)		(reference)	
30–44		1,187	465	38.7	0.76	0.54–1.07	0.88	0.61–1.28
45–59		4,337	1,619	36.8	0.71*	0.51–0.98	0.76	0.53–1.08
60–74		9,841	3,491	35.2	0.66*	0.48–0.90	0.66*	0.47–0.95
≥75		3,996	1,337	33	0.60*	0.43–0.83	0.57*	0.39–0.82
Years of schooling, (mean/sd)‡			9.4/2.0		1.03*	1.01–1.04	1.01	1.00–1.03
*BMI*								
<25 kg/m^2^		4,035	1,639	40.6	(reference)		(reference)	
25–29.9 kg/m^2^		8,200	3,027	36.3	0.83*	0.77–0.90	0.85*	0.77–0.93
30+ kg/m^2^		7,019	2,237	31.4	0.67*	0.61–0.73	0.64*	0.57–0.70
Current non-smoker		15,881	5,689	35.3	(reference)		(reference)	
Current smoker		2,752	1,005	36.5	1.05	0.96–1.15	0.98	0.88–1.09
Physical activity ≥2 hours/week		10,883	4,017	36.5	(reference)		(reference)	
Physical activity <2 hours/week		5,271	1,843	34.4	0.91*	0.85–0.98	0.94	0.86–1.01
*Comorbidities*								
no CHD		14,821	5,037	33.4	(reference)		(reference)	
CHD		4,708	1,952	41.7	1.42*	1.32–1.53	1.25*	1.13–1.39
no Hyperlipidemia		10,035	3,572	35.1	(reference)		(reference)	
Hyperlipidemia		9,494	3,417	35.7	1.03	0.97–1.10	0.97	0.90–1.05
no Diabetes mellitus		13,757	4,969	35.7	(reference)		(reference)	
Diabetes mellitus		5,772	2,020	34.7	0.96	0.89–1.02	0.99	0.91–1.08
no Myocardial infarction		18,000	6,242	34.2	(reference)		(reference)	
Myocardial infarction		1,529	747	48.6	1.82*	1.63–2.03	1.76*	1.50–2.05
no Antihypertensive treatment		1,533	458	29.4	(reference)		(reference)	
Antihypertensive treatment		17,236	6,296	36.2	1.36*	1.21–1.53	1.41*	1.23–1.61

N = 20,164, %w = weighted percentages; OR = Odds Ratio estimated by logistic regression; 95% CI = 95% confidence interval.

# unadjusted OR;

† adjusted OR from multivariate analyses;

* significant on 5% level.

‡ OR for increase of 1 year.

## Discussion

Prevalence of hypertension was dependent on the definition used, and was lowest for HTN_doc_ and highest for HTN_NHANES_. Comparison of diagnosis and treatment rates and of the proportion with adequate control among patients in the DETECT and HYDRA studies showed only a small improvement in treatment and control rates between 2001 and 2003 and a slight decline in diagnosis rates. The comorbidities of CHD and previous myocardial infarction were associated with pharmacotherapy and adequate blood pressure control, whereas for patients with other comorbidities such as DM and hyperlipidemia control was less likely.

### Prevalence of Hypertension

Different definitions of hypertension revealed remarkable differences in prevalence rates. The prevalence rates were lowest for HTN_doc_ and HTN_doc,pat_ and highest for HTN_NHANES_ and HTN_doc,pat,bp_ (56.0% and 55.2%, respectively). This shows that a considerable fraction of people with hypertension had not been diagnosed by physicians. As a consequence treatment and control rates of HTN_NHANES_ based cases are lower in comparison with treatment and control rates based on physician based diagnoses.

Only one study (HYDRA) which is similar to the current one uses the same definitions for hypertension and for the calculation of the prevalence, diagnosis, treatment and control of hypertension [3]. Prevalence rates of hypertension in this study were slightly higher over all groups for HTN_doc_ with 38.8% and slightly lower for HTN_NHANES_ with 50.4%. HTN_doc,pat_ with 39.5% and HTN_doc,pat,bp_ with 52.4% were almost in the same range as in the DETECT study. The total prevalence of hypertension based only on physician’s assigned diagnosis should be considered with caution and these results were found in both studies. The degree of underestimation is more pronounced for the younger patients, but is still notable in the older age groups. Our results for the prevalence rate of hypertension are in accordance with a population-based study for Germany (German National Health Interview and Examination Survey 1998) with prevalence rates only slightly lower if applying NHANES definition criteria in this study (53.5% for men and 43.6% for women) [2]. These results suggest that a substantial proportion of people in primary care with hypertension according to NHANES definition criteria are not diagnosed as hypertensive by physicians in Germany.

In a cohort study with original and offspring cohort members of the Framingham Heart Study, the prevalence rate of hypertension according to the NHANES criteria was 27.3% in participants younger than 60 years of age, 63.0% in participants between 60 and 79 years of age and 74.0% in participants above 80 years of age [24]. These age-specific prevalence rates are lower than for the DETECT study (79.0% for participants between age 60–74 and 86.8% for participants above age 75). One study identified international differences in prevalence, treatment and control rates of hypertension in six different countries (France, Germany, Italy, Spain, UK, US) and used the CardioMonitor Surveys for the year 2004 [25]. In that study, the prevalence of hypertension was determined according to the physician’s diagnosis, which is identical to our HTN_doc_ definition. Between these six countries there was a range of prevalence rates of hypertension between 78% in UK and 90% in Italy, the prevalence in Germany being 86%. The surprisingly high prevalence rates of hypertension in comparison to the DETECT and HYDRA study can be explained partly by the fact, that these surveys were visit-based and not population-based. Physician sampling was based on a quota criterion – each physician had a minimum of 15 patients per week with a cardiovascular disease to be included in the respective countries. Sicker patients were oversampled and as a consequence a selection problem with a high proportion of patients with comorbidities existed. The high prevalence rates for hyperlipidemia with 49% and for DM with 29% in the German CardioMonitor survey are in accordance with this interpretation. The prevalence rates in the DETECT study were much lower for hyperlipidemia with 29.5% and for DM with 12.1%.

### Diagnosis, Treatment and Control Rates of Hypertension

On the one hand, there is a decrease in the proportion of patients diagnosed by physicians in the DETECT study in comparison to the HYDRA study. On the other hand, age-specific treatment and control rates were slightly higher. This means that, despite continuous efforts to improve management of hypertension which have included the publication of the HYDRA results, intense continuous medical education (CME) and intensive training programs, there is only a slight improvement of treatment and control and even a decline in the diagnosis rate.

The WHO-Monica-Augsburg study, conducted in 1994/95, has also reported diagnosis, treatment and control rates which have been in a lower range [4]. In contrast to our study, diagnosis of hypertension was defined as reporting a prior diagnosis of hypertension by the participants instead of using the physicians’ diagnosis. Diagnosis rate was about 60% for women and 50% for men in 1994/95 and similar to own results in the DETECT study. In comparison with the DETECT study, treatment rates were lower (23% in men and 33% in women) and also control rates were lower (7% in men and 13% in women). Further German studies, for example, the German National Health Interview and Examination Survey in 1998 [16], have the limitation of using another definition for diagnosis of hypertension (awareness) or the study has only been conducted in certain parts of Germany, as the Study of Health in Pomerania (SHIP) [26].

Higher control rates of hypertension were reported in the USA compared to those in Germany [22], [27]. In the Framingham Heart Study treatment rates were 68.9% across the age groups [24]. Overall, the rates of control were 32.4% in participants with hypertension and 47.0% in treated participants. The estimated sex-specific rate of control was 38% for women and 23% for men.

### Factors Associated with Pharmacotherapy

DETECT also allowed for the evaluation of the impact of patient characteristics as determinants of pharmacotherapy. Patients with higher age and BMI and comorbidities such as CHD, DM, myocardial infarction, hyperlipidemia showed a higher probability of receiving pharmacotherapy, in agreement with guidelines. These results are partially different from those of the ESTHER study, which is a cohort study conducted in a primary care setting in the German Federal State of Saarland [28]. The analyzed sample were hypertensive patients with DM. Similar results for predictors of receiving pharmacotherapy as in the DETECT study were obtained for higher age and BMI, female sex, CHD and current non-smoking. Physical activity and hyperlipidemia had no influence which is in contrast to the DETECT results. A study in the USA of members of the National Health and Human Services Employees Union showed a higher likelihood of receiving pharmacotherapy in participants with a higher age and BMI than in the DETECT and ESTHER study [29]. Sex had, in contrast to the German studies, no influence.

### Factors Associated with Blood Pressure Control

In the DETECT study female sex, a lower BMI, CHD, previous myocardial infarction and antihypertensive medication were associated with adequate blood pressure control. This is in accordance with guideline recommendations that in patients with comorbidities such as CHD or previous myocardial infarction blood pressure control is of high importance. These results are in contrast to the results of the ESTHER study. In this study the only significant negative predictor of an adequate blood pressure in patients with both DM and hypertension was higher age. The difference between DETECT and ESTHER could be explained first by the fact that hypertensive patients with DM are a special subpopulation of hypertensive patients, and second, by choosing a different cut-off point for adequate blood pressure control with a systolic BP of 130 mmHg and a diastolic BP of 85 mmHg in the ESTHER study.

Results for the prediction of adequate blood pressure control in the DETECT study are also in part in agreement with data for hypertensive patients with DM treated in primary care clinics in the USA [30]. In a retrospective study a higher age was a significant negative predictor for adequate blood pressure control. A history of CHD and also at least one annual visit to a subspecialist had a significant positive influence. In contrast to the results of the DETECT study, no influence of BMI and gender were found and also smoking and physical activity had no influence. Two further studies in the USA estimating control rates had differing results. In the first only female sex and the number of antihypertensive drugs days had a positive significant influence on control of hypertension, whereas higher age, race and a higher BMI played no significant role [29]. The National Health and Nutrition Examination Survey (NHANES) showed that an age of at least 65 years, male sex, not having visited a physician during the last 12 months had a negative predictive value for an adequate blood pressure control in patients aware of their hypertension [31]. Education, family income and current smoking status had no influence on blood pressure control. Comparing the results from all these studies with the DETECT study shows that a comorbidity such as CHD leads to a higher probability of adequate control, although other potentially remediable factors such as smoking status or physical activity show no association.

As a first limitation of the DETECT study it should be mentioned that blood pressure measurement was only done once, and not repeatedly, under primary care conditions. For a high degree of certainty about diagnosis of hypertension different methods and using the average of several measurements are recommended [23]. A single measurement could increase the prevalence of hypertension according to the HTN_NHANES_ definition, because the phenomenon of white coat hypertension could subsequently lead to an underestimation of treatment rates and blood pressure control. In the original study which defined the HTN_NHANES_ criteria for measuring prevalence in the US, two sets of measurements during two different visits had been taken [21]. A second limitation of our analysis is the coding of medical diagnoses DM, CHD and hyperlipidemia: it has been based only on a clinical assessment and has not been validated by objective criteria. A third limitation is that a central laboratory was only used in a subset of patients while in other patients values were taken from the patientś records. A fourth limitation is that the time interval between the acquisition of laboratory values and completing the questionnaire was not comparable for all patients, because it was not fixed for all patients. A further limitation was that for estimation of treatment rates of hypertension only pharmacological interventions were considered and no non-pharmacological interventions were taken into account, so that the treatment rates would be higher if non-pharmacological interventions could also have been considered. Estimation of control rates would have been lower if recommended BP values in the guidelines for patients with comorbidities including hyperlipidemia or DM would have been considered.

### Conclusions

Differing definitions of hypertension exist and as a consequence the prevalence rates of hypertension vary accordingly in the DETECT and HYDRA studies. A comparison of the rates of treated and controlled hypertension in the DETECT study with the HYDRA study show indications of small improvements in proportions of patients adequately controlled, although levels of control remain low. With respect to proportions of people diagnosed, there is even a decline. Hypertensive patients with risk factors such as obesity and associated comorbidities such as CHD, hyperlipidemia and DM were more likely to receive antihypertensive drugs in accordance with guidelines. A higher rate of adequate blood pressure control was achieved in patients with obesity and CHD, but not those with DM or hyperlipidemia.
